# Impact of climate change on formation of nitrogenous disinfection by-products. Part II: water blooming and enrichment by humic substances

**DOI:** 10.1007/s11356-024-32960-4

**Published:** 2024-03-28

**Authors:** Argyri Kozari, Spyros Gkellis, Dimitra Voutsa

**Affiliations:** 1 Environmental Pollution Control Laboratory, School of Chemistry, Aristotle University, 541 24 Thessaloniki, Greece; 2 Department of Botany, School of Biology, Aristotle University, 541 24 Thessaloniki, Greece

**Keywords:** Climate change, Disinfection byproducts, Water blooming, Humic acids, N-DBPs, AOM, DOM

## Abstract

Climate change affects the concentration and characteristics of dissolved organic matter (DOM) in surface water. The changes in composition of DOM have many implications to drinking water quality, especially in the case of formation of disinfection by-products (DBPs). The aim of this study was to investigate the formation of nitrogenous DBPs (N-DBPs) during chlorination and chloramination, caused by the alternation of surface water’s DOM driven by climate change. For this reason, two different cases were examined: (a) rise of algal organic matter (AOM) due to water blooming and (b) water enrichment by humic substances. The target compounds were haloacetonitriles (HANs), haloacetamides (HAcAms), and halonitromethane (TCNM). The results showed that *Anabaena* appears to be a major precursor for HAcAms and TCNM, while humic acids are precursors for HANs. The results of the mixtures presented the same pattern. During the water blooming case, HAcAms and TCNM formation are in favor, while during water enrichment by humic substances case, HANs is the N-DBP group with higher formation yield. Cloraminated samples presented higher values of cytotoxicity and genotoxicity compared to the chlorinated.

**Graphical Abstract:**

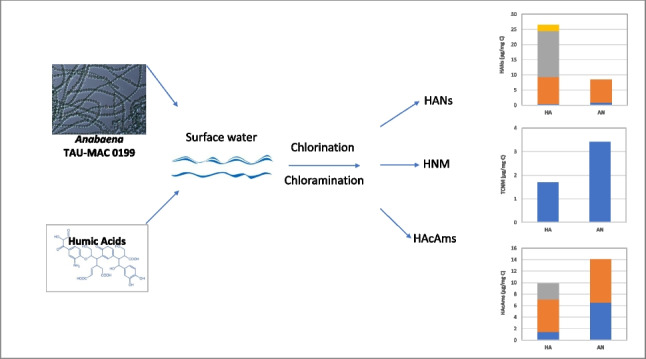

**Supplementary Information:**

The online version contains supplementary material available at 10.1007/s11356-024-32960-4.

## Introduction

Climate change can lead to significant disturbances in hydrological cycle. Increasing temperatures, changing patterns of precipitation, and frequent extreme events such as floods and droughts have significant impacts on water cycle. Shifts in hydroclimatic regimes represent an emerging challenge for efficient management of water resources with respect to water availability and quality (Paerl and Barnard 2020; IPCC 2022; Ryberg and Chanat 2022; Elhabashy et al. 2023). Climate disturbances in water cycle seriously threaten the achievement of water goal SDG6 of the “2030 Agenda for Sustainable Development”, as well as other sustainable development sub-goals linking to this goal.

Hydroclimatic and biogeochemical processes control organic carbon in surface waters. Organic carbon dynamics in surface waters are affected by both terrestrial and in-stream processes. Changes of concentration and/or changes in characteristics of dissolved organic matter (DOM) are important key factors for water quality (Du et al. 2019; 2020; Sharma et al. 2023). Over the last 30 years, an increase of DOM in surface waters has been reported worldwide (Lipczynska-Kochany 2018) and is expected to become more intense in the future due to climate change. In surface waters, humic substances represent the main fraction of DOM (Stergiadi et al. 2016). This fraction has the ability of solar radiation’s absorbance, causing microbial interactions that affect aquatic organisms (Paul et al. 2006). The rise in temperature leads to an increase in the production of DOM into water bodies, while flooding events contribute to the excess export of DOM from soil to surface waters through runoff (Lipczynska-Kochany 2018; Travnik et al. 2009). These factors lead to the rise of humic substance content in the water (Canellas et al. 2015). Moreover, extended dry periods coupled with high temperatures and then following extreme storms increase algal growth and the risk of harmful blooms (Piccolroaz et al. 2020; Nijhawan and Howard 2022; Mejean et al. 2014) and consequently the rise of algal organic matter (AOM) into water bodies.

The increase in concentration as well as changes in composition of DOM pose implications to water treatment (Delpla et al. 2009), as surface waters are major sources of drinking water. Thus, it is a challenge for the conventional treatment processes to achieve the desired quality of drinking water (Howard et al. 2016; Sjerps et al. 2017; Yang et al. 2018; Liu et al. 2020). During disinfection processes, DOM’s precursors react with chlorine, leading to the formation of disinfection by-products (DBPs). Different sources of DOM may affect not only the concentrations but also the profile of DBPs and consequently the overall risk for human health (Chang et al. 2013; Hong et al. 2013; Doederer et al. 2014; Bond et al. 2015; Benson et al. 2017; Chen et al. 2019; Kozari et al. 2020; Wang et al. 2021; Wu et al. 2022).

The presence of DBPs in drinking water is a great matter of concern for public health, as many of them present high values of toxicity and have carcinogenic and mutagenic activity (Benson et al. 2017; Plewa et al. 2017). Due to the potential health effects of DBPs, the possible impact of climate on DBP formation is currently of important concern (Delpla et al. 2016; Delpla and Rodriguez 2017; Cool et al. 2019; Valdivia-Garcia et al. 2019; Ma et al. 2022; Kozari and Voutsa 2023).

Most studies have been focused on common and regulated DBPs, such as trihalomethanes (THMs) and haloacetic acids (HAAs) (Directive EU, 2020). However, there are emerging groups of DBPs that present higher toxicity than the regulated (Plewa et al. 2017; 2008). This category includes the nitrogenous disinfection by-products (N-DBPs).

Following our previous work Kozari and Voutsa (2023), this study focuses on the possible impact of DOM, derived from different sources, on the formation of N-DBPs. For this reason, two different cases driven by climate change under water disinfection were chosen: (a) water blooming caused by cyanobacteria *Anabaena sp.* and (b) water enrichment by humic substances. River water, humic acids, algal organic matter, and mixtures at different gradients were subjected to chlorination and chloramination under different experimental conditions to investigate the formation of N-DBPs.

## Materials and methods

### Chemicals and materials

All chemical solutions were of analytical grade or better. Milli-Q water (Simplicity UV Ultrapure Water System, Millipore, Molsheim, France) was used for the preparation of aquatic solutions. The analytical standard solution for haloacetonitriles (trichloroacetonitrile, TCAN; dichloroacetonitrile, DCAN; bromochloroacetonitrile, BCAN; and dibromoacetontrile, DBAN) and chloropicrin was EPA 551B Halogenated Volatiles Mix, 2000 μg/ml in MTBE by Sigma–Aldrich Ltd. MtBE, Na_2_HPO_4_, KH_2_PO_4_, and NH_4_Cl were purchased from Sigma–Aldrich Ltd. too. The analytical standards for haloacetamides (chloroacetamide, CAcAm; dichloroacetamide, DCAcAm; and bromoacetamide, BAcAm) were purchased from Alfa–Aesar Ltd. 1,2-dibromopropane was purchased from Chem. Service (West Chester), NaOCl (available chlorine 10% w/v) from Panreac, Ascorbic acid from Chem Lab, and humic acids (technical) were provided by Fluka™.

### Climate change cases

This study focuses on the possible impacts of climate change on Aliakmon River’s DOM, the main source of drinking water in the city of Thessaloniki. About 150,000 m^3^ of water from the Aliakmon River is used per day to provide drinking water to almost 1,000,000 inhabitants. Details about the study area are published in previous work (Kozari and Voutsa 2023).

The physicochemical parameters of the Aliakmon River through recent years have been published by Papageorgiou et al. (2014; 2016). The concentrations of dissolved organic carbon (DOC) ranged from 1.5 to 3.0 mg C/L. Differences have been observed with respect to fluorescence intensity that exhibited the highest intensity during warmer months, specific absorbance (SUVA), and hydrophobic/hydrophilic fractions of NOM.

These differences could be attributed to different contribution of autochthonous or allochthonous organic matter. Thus, in this study, we investigate the impact of the increase of algal and humic acid content.

#### Water blooming case

Aliakmon River resides in Northern Greece. Streams, artificial lakes, and dams are linked with this river. Through the years, phytoplankton masses and various algae species have been observed in Aliakmon’s water (Chrisostomou et al. 2009; Gkelis et al. 2015a, b; Demertzioglou et al. 2022). In order to study the case of water blooming in the formation of N-DBPs, the cyanobacterium strain *Anabaena* cf*. oscillarioides* was chosen.

*Anabaena* has been found in the surface water of the Aliakmon River (Gkelis et al. 2015a, b; Montesanto et al. 1999) *Anabaena*/*Dolichospermum* is one of the most frequent cyanobacteria forming algal blooms in Greece (Gkelis et al. 2015a, b) and worldwide (Ibelings et al. 2021). The strain *Anabaena* cf*. oscillarioides* TAU-MAC 0199 was isolated from Lake Paralimni (Gkelis et al. 2015b), and it is held in the Thessaloniki Aristotle University Microalgae and Cyanobacteria (TAU-MAC) Culture Collection (Gkelis and Panou 2016) and is a non-toxic planktic cyanobacterium (Gkelis et al. 2019). The strain was cultured in BG11 medium at 20 ± 2 °C, 20 µmol m^−2^ s^−1^ light intensity, with a rate of 16:8 day/night for 9 weeks at 2-L flasks.

After the cultivation was completed, biomass of *Anabaena* TAU-MAC 0199 was spiked in river water samples from Aliakmon under different proportions in order to achieve 15% (AN 15) and 30% (AN 30) contribution of AOM to the total DOC concentrations of the samples (Table 1).

**Table 1:** Studied regimes and experimental conditions

Climate-driven cases	Studied regimes	Description	Chlorination/chloramination	
			**Dose (mg/L)**	**Contact time (h)**
River	RI	River water	5, 10	24, 72
Water blooming (*Anabaena*)	AN 15	15% contribution of algal organic carbon to DOC of river water	5, 10	24, 72
	AN 30	30% contribution of algal organic carbon to DOC of river water	5, 10	24, 72
Water enrichment by humic substances	HA 15	15% contribution of humic organic carbon to DOC of river water	5, 10	24, 72
	HA 30	30% contribution of humic organic carbon to DOC of river water	5, 10	24, 72

#### Water enrichment by humic substance case

Hydroclimate changes are linked to the enrichment of humic substances in water bodies through flooding and runoff in river watersheds (Du et al. 2019; 2020). In order to study the impact of humic substances in the formation of N-DBPs, river samples from Aliakmon were collected, and then humic acids were spiked at different proportions in order to achieve 15% (HA 15) and 30% (HA 30) contribution of humic acids to the total DOC concentrations of the samples (Table 1).

### Chlor(am)ination experiments

Compared to formation potential tests, simulated distribution system tests (SDS) predict the possible formation of DBPs better, as the experimental conditions are closer to real water treatment conditions (Sfynia et al. 2017; Kanan and Karanfil 2020). Thus, SDS was chosen in our project to study the formation of N-DBPs.

As disinfection techniques, chlorination and chloramination were chosen. The disinfection experiments were employed in samples/mixtures of river water (RI) from Aliakmon River, *Anabaena sp*. (AN) and humic acids (HA) (Table 1). The chlorination agent was sodium hypochlorite solution, while the chloramination agent was monochloramine solution. The details of disinfection experiments are given to Kozari and Voutsa (2023). In summary, the conditions of the experiments were different contact times (24, 72 h) and different disinfectant doses (5, 10 mg/L as Cl_2_) under the same temperature (20.0 ± 1.0 °C) and pH (7.8 ± 0.2), and all the samples were kept free of headspace. The range of chlorine residual was 0.2–2 mg/L. NH_4_Cl terminated the chlorination process, and ascorbic acid was used for the termination of the chloramination process.

### Analysis of Nitrogenous DBPs

Seven N-DBPs (4 haloacetonitriles, 3 haloacetamides, and 1 halonitromethane) were the target DBPs, and they were extracted from the samples by liquid–liquid extraction with MtBE, according to the modified US EPA Method 551.1 (U.S. EPA 1995; Sfynia et al. 2020). For the enhancement of the ionic strength and the better separation of the phases, Na_2_SO_4_ was added before the extraction. After the extraction, the internal standard (1,2-dibromopropane) was added to the organic phase. The analysis of the studied N-DBPs was held by GC/ECD (Trace GC Ultra, Thermo Scientific). The details about the characteristics of the chromatographic column and the temperature program are provided in Kozari and Voutsa (2023). All the experiments were employed in triplicates, and the relative standard deviation was < 15%. The quality parameters are shown in Table S1.

### Measurements of DOC, N-species, and residual chlorine

Dissolved organic carbon (DOC) was determined by a TOC-Vcsh analyzer (Shimadzu). For the measurement of N-species (NO_2_^−^, NO_3_^−^, NH_4_^+^, ΤΟΝ, and TN), we based on the Standard methods for the examination of water and wastewater (A.P.H.A., 2017, 23rd Ed.), using a spectrophotometer (Hitachi U-2001). The absorbance of UV at 254 nm was held at the same spectrophotometer. The results of DOC, N-species, and SUVA values are shown in Table S2. Free residual chlorine was measured by HACH spectrophotometer (model DR 3900) according to the method 8021 and total chlorine by the HACH method 8167 (HACH, 2014).

### Incorporation of bromine

For the calculation of the incorporation of bromine, the incorporation factor (BIF) was used. The BIF is the ratio of bromine’s moles to the total incorporated halogen’s moles of the different classes of disinfection by-products species. Thus, Eq. (1) was used for the calculation of incorporation of bromine in the class of HANs, while Eq. (2) for the HAcAms.

1 $${\mathrm{BIF}}_{\mathrm{HANS}}=\frac{1\mathrm{BCAN}+2\mathrm{DBAN}}{\mathrm\Sigma\mathrm H\mathrm A\mathrm N\mathrm s}$$

2 $${\mathrm{BIF}}_{\mathrm{HAcAms}}=\frac{1\mathrm{BAcAm}}{\mathrm\Sigma\mathrm H\mathrm A\mathrm c\mathrm A\mathrm m\mathrm s}$$

### Cytotoxicity and genotoxicity assessment

Potential cytotoxicity and genotoxicity οf the samples were calculated. The calculation of cytotoxicity was based on the molar concentrations of each N-DBP and their respective LC_50_ values, while genotoxicity was based on published potencies. These cyto- and geno-values are results from assays of Chinese hamster ovary (CHO) cells (Wagner and Plewa 2017). Many researchers use the above calculations for the assessment of the toxicity of disinfection by-products (Liu et al. 2018; Postigo et al. 2018; Ersan et al. 2019; Kozari et al. 2020).

### Statistical analysis

For the statistical analysis, Microsoft Excel and IBM SPSS Statistics software were used. The performed correlation analysis was based on Spearman’s rho.

## Results and discussion

### Water blooming case

#### Chlor(am)ination of algal organic matter

**Fig. 1:**
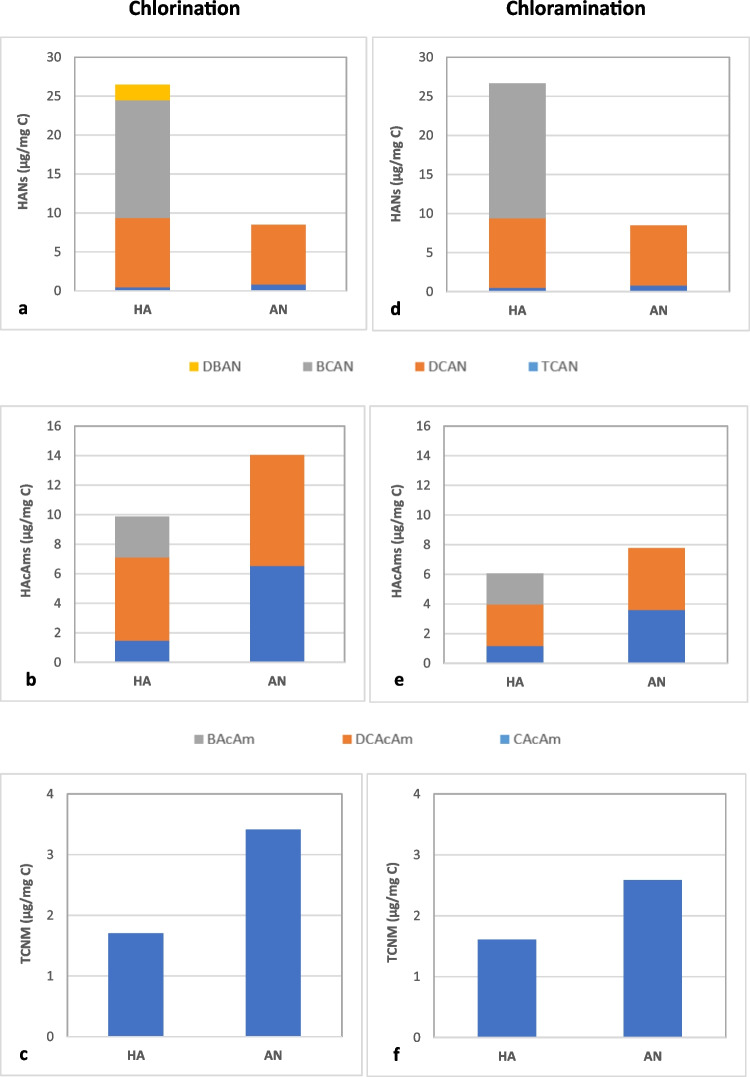
Formation of HANs, HAcAms, and TCNM upon chlorination (**a**, **b**, **c**) and chloramination (**d**, **e**, **f**) of humic acids (HA) and *Anabaena* (AN) (chlor(am)ination conditions: dose 10 mg/L, contact time 72 h)

The impact of water blooming in Aliakmon River was studied focusing on cyanobacteria *Anabaena*. For this reason, the intracellular matter of *Anabaena* was chlorinated and chloraminated under different doses (5, 10 mg/L) and contact times (24, 72 h). The formation yields of the studied DBPs are shown in Fig. 1. Halocetamides (HAcAms) were the major N-DBP class in both disinfection processes. Brominated species were not detected at all. Chlorination experiments led to a higher formation yield of N-DBPs (mainly HAcAms and TCNM) compared to the chloramination process.

These results are in accordance with other studies indicating *Anabaena* as an important precursor to the formation of HAcAms and TCNM (Liao et al. 2022; Zhang et al. 2021; Sfynia et al. 2020; W.H.O. 2003). The formation of these N-DBPs is highly dependent on the presence of protein-like material in water. In general, HAcAms formation mechanisms remain unclear, and the precursors of HAcAms in natural waters still need to be characterized. HAcAms were first reported to be intermediate products of HANs hydrolysis, but also there are studies indicating that the mechanism of HAcAms formation can be independent from that of HANs (Le Roux et al. 2016). There is evidence that the hydrophilic fraction isolated from an algal-impacted water enriched in protein-like organic matter exhibits the highest HAcAms formation during chlorination and chloramination processes (Chu et al. 2010; Chorus and Bartram 1999). The abundance of proteinaceous-like materials in AOM contributes to a high DON/DOC ratio but low SUVA values (Wang et al. 2021). In our case, the ratio DON/DOC was 0.32, while the SUVA value was at 0.894 L/mgC·m. Moreover, according to Chuang et al. (2015), the formation of some intermediate compounds is faster during chlorination compared to chloramination, playing the role of an important precursors’ pool to the formation of the final DBPs.

#### Chlor(am)ination of algal regimes

Two different regimes were used: 15% contribution of *Anabaena sp*. organic matter into river water’s DOC (AN 15) and 30% contribution of *Anabaena* organic matter into river water’s DOC (AN 30). Chlorination and chloramination experiments were conducted under the above conditions, and the specific formation yields (expressed as μg/mgC) of haloacetonitriles, haloacetamides, and nitromethane are shown in Figure S1.

##### Haloacetonitriles (HANs)

**Fig. 2:**
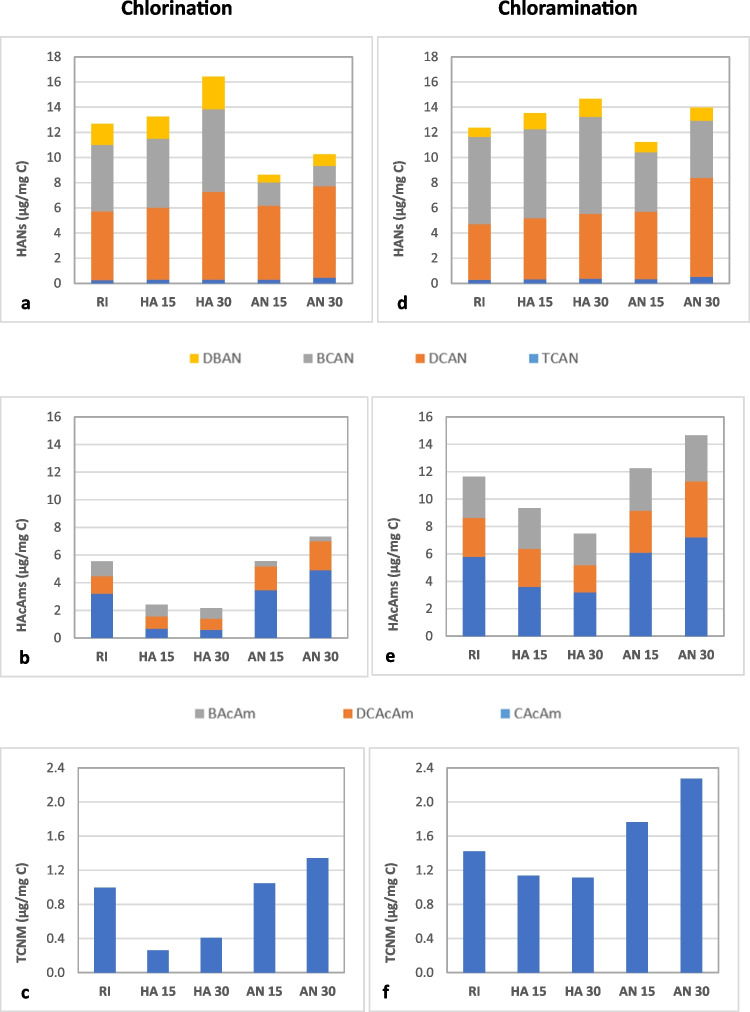
Formation of HANs, HAcAms, and TCNM under different regimes of humic acids (HA) and *Anabaena* (AN) to the river water (RI): chlorination experiments (**a**, **b**, **c**) and chloramination experiments (**d**, **e**, **f**) (chlor(am)ination conditions: dose 10 mg/L, contact time 72 h)

**Fig. 3:**
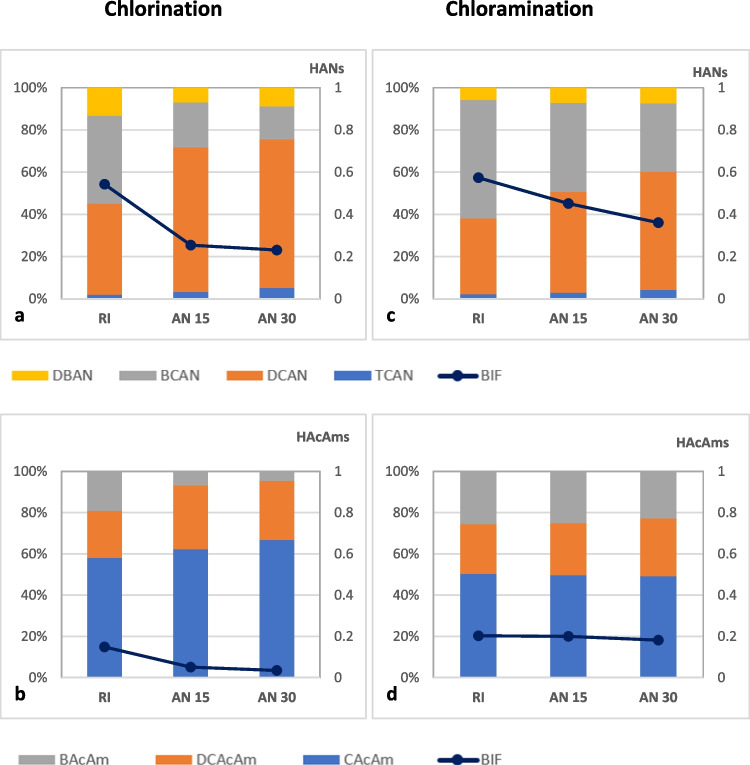
Profile of HANs and HAcAms and their respective BIF during chlorination (**a**, **b**) and chloramination (**c**, **d**) under different regimes of *Anabaena* (AN) to the river water (RI) (dose 10 mg/L, contact time 72 h)

The concentrations of the sum of four haloacetonitriles (TCAN, DCAN, BCAN, and DBAN) under chlor(am)ination are shown in Fig. 2a, d. Each compound’s relative contribution and BIF are presented in Fig. 3a, c.

The presence of AOM from *Anabaena* in river water did not have an impact on the formation of HANs during chloramination. However, a significant reduction of HANs was observed during chlorination (Fig. 2a, d). At the same time, the profile of HANs is different in both disinfection processes as the ratio of *Anabaena* to river water grew. Specifically, in river water samples (RI), DCAN and BCAN were the major haloacetonitriles upon chlor(am)ination, followed by DBAN, whereas TCAN was in barely detectable levels. This could be attributed to the fact that dihalogenated nitriles are reported to be more stable than trihalogenated (Watson et al. 2012). The contribution of *Anabaena* enhanced the formation of DCAN (up to 50%) at the expense of the brominated HANs (Fig. 3a,c). Subsequently, a reduction of BIF values also has been observed. TCAN and DCAN correlate significantly with DOC and N-species (NO_2 _^−^, NO_3_^−^, NH_4_^+^, TON, TN), while BCAN and DBAN present a negative correlation with the above parameters.

##### Haloacetamides (HAcAms)

The concentrations of the sum of haloacetamides (CAcAm, DCAcAm, and BAcAm) under chlor(am)ination are shown in Fig. 2b, e. Each compound’s relative contribution and BIF are presented in Fig. 3b, d.

As the content of *Anabaena* in river water increases, the formation of HAcAms is enhanced during chlor(am)ination. This could be attributed to the presence of precursors, such as protein-like materials and amino acids in AOM (Liao et al. 2022; Sfynia et al. 2020; Zhang et al. 2021; Wang et al. 2021; Wu et al. 2022). The content of TON in these regimes is higher than TON of river samples (RI) (Table S2). However, differences in the HAcAms profile were observed. In the case of chlorination, an increase of CAcAm was evident (20%) (Fig. 3b), which becomes the major HAcAm, while BAcAm becomes the acetamide formed less. In the case of chloramination, the sum of HAcAms exhibited higher concentrations compared to chlorination (Fig. 2b, e). However, both river water and river water—*Anabaena—*presented similar profiles of HAcAms (Fig. 3d). Using Spearman’s correlation, HAcAms present a negative correlation with SUVA. CAcAm and DCAcAm correlate significantly with DOC, and N-species (NO_2_^−^, NO_3_^−^, NH_4_^+^, TON, TN), while BAcAm presents a negative correlation with the above parameters.

##### Halonitromethane (HNM)

Chloropicrin (TCNM) is the major representative compound of halonitromethanes. According to WHO (2003), the major precursors that contribute to the formation of TCNM are amino acids and nitrophenols. At the same time, high concentrations of NO_3_^−^ and NH_4_^+^ in surface water contribute to the enhancement of the TCNM’s formation during water treatment (Wang et al. 2021; Ding et al. 2018).

In our study, river water presented low formation of TCNM (Fig. 2c, f). As the content of *Anabaena* increases, TCNM formation is promoted in both chlorination and chloramination. Chloramination experiments gave a higher formation of TCNM. Using Spearman’s rho, TCNM correlates significantly with DOC, NO_2_^−^, NO_3_^−^, NH_4_^+^, TON, and TN and presents a negative correlation with SUVA.

### Water enrichment by humic substances case

#### Chlor(am)ination of humic acids

The impact of humic substances in the formation of N-DBPs was studied. For this reason, a solution of humic acids was chlorinated and chloraminated under different doses (5, 10 mg/L) and contact times (24, 72 h). The formation yield of the studied DBPs is shown in Fig. 1.

Haloacetonitriles (HANs) were the major formed N-DBP class, and BCAN was the major HAN. Humic acids can be an important precursor to the formation of HANs. According to Reckhow et al. (1990), a positive correlation was found between humic substances’ nitrogen content and their tendency to form HANs upon chlorination. At the same time, the aromatic moieties in NOM seem to play an important role in the formation of HANs (Le Roux et al. 2016). SUVA values of NOM are high because of humic-like substances (Wang et al. 2021). In our case, the ratio DON/DOC was 0.08, while the SUVA value was at 3.123 L/mgC·m. In general, the reaction of natural organic matter with chlorine initially leads to the formation of nonhalogenated aromatic DBPs, which are unstable compounds, and they tend to be transformed into toxic halogenated aromatic DBPs that further react with chlorine to generate commonly known aliphatic DBPs (Jiang et al. 2020; Han et al. 2021). Nihemaiti et al. (2017) reported the formation of N-DBPs upon chloramination of nitrogenous and non-nitrogenous aromatic compounds through the formation of nitrogenous heterocyclic intermediates, whereas Zhang et al. (2022) reported both chloramine and nitrogen compounds are possible sources of nitrogen for N-DBPs.

#### Chlor(am)ination of humic regimes

Two different regimes were examined: 15% contribution of humic acids to river water’s DOC (HA 15) and 30% contribution of humic acids to river water’s DOC (HA 30). Chlorination and chloramination experiments were conducted under the above conditions. The specific formation yields (expressed as μg/mgC) of haloacetonitriles, haloacetamides, and halonitromethane are shown in Figure S2.

##### Haloacetonitriles (HANs)

**Fig. 4:**
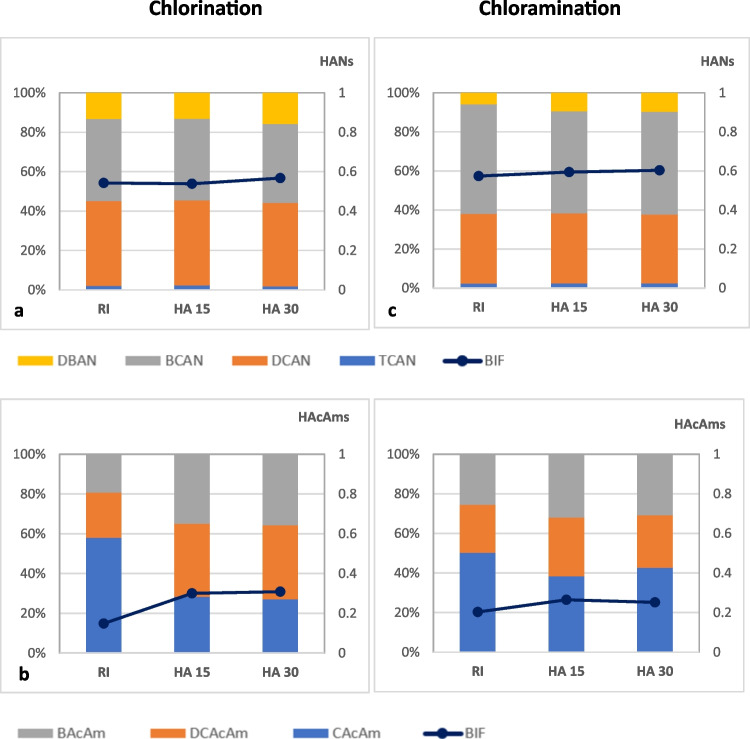
Profile of HANs and HAcAms and their respective BIF during chlorination (**a**, **b**) and chloramination (**c**, **d**) under different regimes of humic acids (HA) to the river water (RI) (dose 10 mg/L, contact time 72 h)

The concentrations of the sum of the haloacetonitriles are shown in Fig. 2a, d. Each compound’s relative contribution and BIF are presented in Fig. 4a, c.

An increasing trend of HANs formation was observed in line with humic acid contribution rising in river water’s DOC. The major haloacetonitriles in river samples (RI) both in chlorination and chloramination were DCAN and BCAN. As the ratio of humic acids increased, the formation of DCAN remained at the same levels, while the formation of DBAN was slightly enhanced and a slight reduction of BCAN formation was observed (Fig. 4a, c). BIF values remained at the same level, indicating that no more bromide could be incorporated further.

In this case, HANs present a strong correlation with SUVA. TCAN and DCAN correlate significantly with DOC and N-species (NO_2_^−^, NO_3_^−^, NH_4_^+^, TON, TN) while BCAN and DBAN present a negative correlation with the above parameters.

##### Haloacetamides (HAcAms)

The concentrations of the haloacetamides are shown in Fig. 2b, e. Each compound’s relative contribution and BIF are presented in Fig. 4b, d.

The ongoing rise of humic acids in river samples contributed to the decrease of HAcAms formation in both disinfection processes. In river samples (RI), the major acetamide was CAcAm. As the content of humic acids in the river grows, the formation of DCAcAm and BAcAm is promoted. In both disinfection processes, the BIF values slightly increase, indicating a small incorporation of bromide, resulting in a higher formation of BAcAm. In this case, HAcAms present a negative correlation with DOC, SUVA, and N-species (NO_2_^−^, NO_3_^−^, NH_4_^+^, TON, TN).

##### Halonitromethane (HNM)

In our experiments, TCNM formation is reduced as humic acids rise into river water (Fig. 2c, f). Chloramination presented higher formation compared to chlorination. Also, TCNM presents a negative correlation with DOC, SUVA, and N-species (NO_2_^−^, NO_3_^−^, NH_4_^+^, TON, TN).

### Cytotoxicity and genotoxicity assessment

**Fig. 5:**
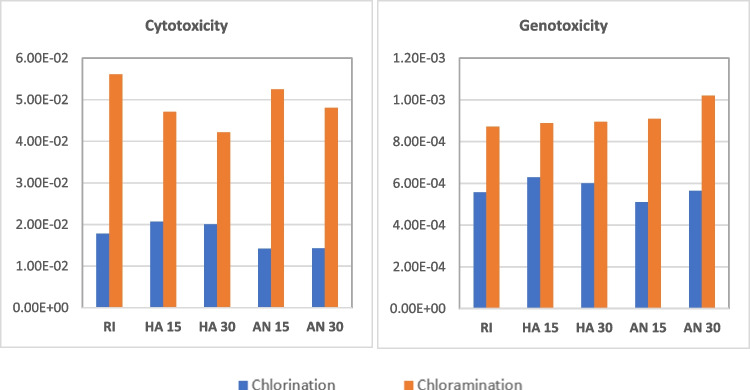
Cytotoxicity and Genotoxicity of the samples during chlorination and chloramination (dose: 10 mg/L, contact time 72 h)

The calculated cytotoxicity and genotoxicity of samples are shown in Fig. 5. Chloramination resulted in higher cytotoxicity and genotoxicity values in all cases compared to chlorination results.

In the case of water blooming, as the content of *Anabaena* in river water increases, the formation of HAcAms is in favor, leading to the rise of genotoxicity, while cytotoxicity presents a slight reduction. This happens as the genotoxicity of HAcAms appears to be slightly higher compared to the genotoxicity of HANs (Postigo et al. 2018; Wagner and Plewa 2017).

In the case of water enrichment by humic substances, a slight increase in cytotoxicity and genotoxicity is observed but only in chlorinated samples. The rise of genotoxicity is not a result of the formation of HAcAms—as it was in the water blooming case—, because the rise of humic acids into river water leads to limited formation of them. Genotoxicity values can be attributed to the fact that the brominated N-DBPs presented a slightly enhanced formation at the expense of the chlorinated N-DBPs. The ongoing rise of the formation of DBAN and BAcAm, as the content of humic acids increases, leads to the enhancement of genotoxicity. This happens as bromo-NDBPs present higher genotoxicity values compared to chloro-NDBPs (Wagner and Plewa 2017; Richardson et al. 2008).

## Conclusions

This study investigated the possible impact of the alternation of surface water’s DOC driven by climate change scenarios (water blooming and enrichment by humic substances) in the formation of N-DBPs (haloacetonitriles, haloacetamides, and halonitromethane) during chlor(am)ination under different contact time and disinfectant dose. At the same time, a risk assessment of N-DBPs was employed based on published cytotoxicity and genotoxicity values.

Chlorination and chloramination resulted in different formation of N-DBPs. In both processes, the high disinfectant dose led to elevated concentrations of N-DBPs, while during the two contact times, each compound presented different behavior. In the case of water blooming, as *Anabaena* content rises, the formation of HAcAms and TCNM is promoted. On the other hand, HANs present a reduction in their formation. In the case of water enrichment by humic acids, HANs are the major group of N-DBPs formed, while HAcAms present a reduction in their formation. *Anabaena* presented a high ratio of DON/DOC and low SUVA value and appears as a major precursor for the formation of HAcAms and TCNM, while the brominated species are not in favor. Humic acids presented a lower DON/DOC ratio but higher SUVA compared to *Anabaena* and contributed to the formation of HANs. There are no significant changes in cytotoxicity and genotoxicity values between the different matrices, but significant changes have been observed between the two disinfection processes. Chloramination leads to higher cytotoxicity and genotoxicity values compared to chlorination.

## Supplementary Information

Below is the link to the electronic supplementary material.

Supplementary file1 (DOCX 87 KB)

## Data Availability

The authors declare that the data supporting the findings of this study are available within the paper and its Supplementary Information files. Should any raw data files be needed in another format they are available from the corresponding author upon reasonable request.
