# Supplementary material for: Impact of climate change on formation of nitrogenous disinfection by-products. Part II: water blooming and enrichment by humic substances
Source: Environ Sci Pollut Res Int. 2024 Mar 28;33(22):10984–95. doi: 10.1007/s11356-024-32960-4 (PMC13415276; doi:10.1007/s11356-024-32960-4)
Supplement: Supplementary file 1 — Supplementary file1 (DOCX 87 KB) [file 11356_2024_32960_MOESM1_ESM.docx]

Supplementary Document

IMPACT OF CLIMATE CHANGE ON FORMATION OF NITROGENOUS DISINFECTION BY PRODUCTS. PART II: WATER BLOOMING AND ENRICHMENT BY HUMIC SUBSTANCES

Argyri Kozari*, Dimitra Voutsa

**TABLES**

Table S1. Analytes and quality parameters

| **Analytes** | **Abbreviation** | **Chemical formula** | **Recovery (%)** | **RSD**  **( % )** | **LOD (μg/L)** |
| --- | --- | --- | --- | --- | --- |
| Trichloroacetonitrile | TCAN | C_2_Cl_3_N | 98 | 7.3 | 0.0003 |
| Dichloroacetonitrile | DCAN | C_2_HCl_2_N | 92 | 8.6 | 0.0005 |
| Bromochloroacetonitrile | BCAN | C_2_HBrClN | 100 | 3.0 | 0.0002 |
| Dibromoacetonitrile | DBAN | C_2_HBr_2_N | 102 | 5.5 | 0.0001 |
| Chloroacetamide | CAcAm | C_2_H_4_ClNO | 86 | 7.1 | 0.0052 |
| Dichloroacetamide | DCAcAm | C_2_H_3_Cl_2_NO | 91 | 6.4 | 0.0010 |
| Bromoacetamide | BAcAm | C_2_H_4_BrNO | 88 | 4.4 | 0.0028 |
| Trichloronitromethane | TCNM | CCl_3_NO_2_ | 95 | 8.7 | 0.0006 |

Table S2. Mean concentrations of DOC and N-species of the studied regimes

| Type of Samples | DOC  (mgC/L) | NO_2_^-^ (mgN/L) | NO_3_^-^  (mgN/L) | NH_4_^+^ (mgN/L) | TON  (mgN/L) | TN  (mgN/L) | SUVA* (L/mgC^.^m) |
| --- | --- | --- | --- | --- | --- | --- | --- |
| River (RI) | 2.452 | 0.004 | 1.085 | 0,071 | 0.07 | 1.16 | 2.814 |
| AN 15 | 2.659 | 0.006 | 1.880 | 0.295 | 0.18 | 2.41 | 2.482 |
| AN 30 | 3.008 | 0.008 | 2.683 | 0.517 | 0.30 | 3.58 | 2.028 |
| HA 15 | 2.574 | 0.005 | 1.092 | 0.080 | 0.07 | 1.20 | 2.875 |
| HA 30 | 2.900 | 0.007 | 1.110 | 0.092 | 0.06 | 1.26 | 2.931 |

* SUVA = UV_254_ x 100 / DOC

**Chlorination Chloramination**

**
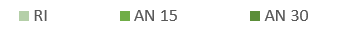
**

Fig. S1: Specific formation yields (μg/mgC, mean±sd) of HANs, HAcAms and TCNM under different regimes of *Anabaena* (AN) to the River water (RI): a,b,c) chlorination experiments and d,e,f) chloramination experiments (chlor(am)ination conditions: doses 5 and 10 mg/L, contact time 24 and 72 h)

**Chlorination Chloramination**

**a**


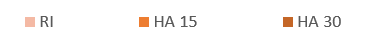


Fig. S2: Specific formation yields (μg/mgC, mean±sd) of HANs, HAcAms and TCNM under different regimes of Humic Acids (HA) to the River water (RI): a,b,c) chlorination experiments and d,e,f) chloramination experiments (chlor(am)ination conditions: doses 5 and 10 mg/L, contact time 24 and 72 h)
